# Genetic analysis of a family affected by congenital myasthenic syndrome due to a Novel mutation in the SLC5A7 gene

**DOI:** 10.1186/s12883-024-03716-x

**Published:** 2024-06-17

**Authors:** Sheng Tian, Huan Sun, Fen-Fang Gao, Kang Zhang, Jing Nan, Mu Niu, Xiao Jia, Gang Xu, Wei Ge

**Affiliations:** 1 Xuzhou Medical University, Xuzhou, China; 2 The Affiliated Hospital of Xuzhou Medical University, Xuzhou Medical College Affiliated Hospital, Xuzhou, China

**Keywords:** Congenital myasthenic syndrome, SLC5A7 gene, Choline transporter, Myasthenia

## Abstract

**Background:**

Mutations in the SLC5A7 gene cause congenital myasthenia, a rare genetic disorder. Mutation points in the SLC5A7 gene differ among individuals and encompass various genetic variations; however, exon deletion variants have yet to be reported in related cases. This study aims to explore the clinical phenotype and genetic traits of a patient with congenital myasthenic syndrome due to SLC5A7 gene variation and those of their family members.

**Case presentation:**

We describe a case of a Chinese male with congenital myasthenic syndrome presenting fluctuating limb weakness. Genetic testing revealed a heterozygous deletion mutation spanning exons 1–9 in the SLC5A7 gene. QPCR confirmed a deletion in exon 9 of the SLC5A7 gene in the patient’s mother and brother. Clinical symptoms of myasthenia improved following treatment with pyridostigmine.

**Conclusion:**

Exons 1, 5, and 9 of the SLC5A7 gene encode the choline transporter’s transmembrane region. Mutations in these exons can impact the stability and plasma membrane levels of the choline transporter. Thus, a heterozygous deletion in exons 1–9 of the SLC5A7 gene could be the pathogenic cause for this patient. In patients exhibiting fluctuating weakness, positive RNS, and seronegativity for myasthenia gravis antibodies, a detailed family history should be considered, and enhanced genetic testing is recommended to determine the cause.

## Background

Congenital myasthenic syndrome (CMS) results from mutations in genes encoding components of the neuromuscular junction (NMJ). Clinical manifestations comprise muscle weakness, hypotonia, severe fatigue, and paroxysmal apnea [1]. Currently, 35 gene variations [2], including CHRNA1, MUSK, CHRND, COLQ, SCN4A, CHAT, SLC5A7, LAMA5, GFPT1, PURA, have been identified in association with CMS. Specifically, the SLC5A7 gene encodes a high-affinity choline transporter (CHT), essential for choline reuptake via the sodium gradient in the NMJ’s presynaptic membrane [3]. CMS cases resulting from SLC5A7 gene mutations are exceedingly rare, with just over 20 cases reported globally and none in China. This paper presents a case of an adult-onset CMS patient, primarily exhibiting fluctuating generalized weakness. Genetic testing identified a heterozygous deletion mutation in exons 1–9 of the SLC5A7 gene. The clinical phenotype and genetic characteristics were analyzed in conjunction with the patient’s family members’ symptoms and existing literature.

## Case report

A 41-year-old male with a high school education, working as a freelancer, was admitted to the Neurology Department in March 2023 for “fluctuating general weakness.” He was a full-term, naturally born infant with no history of intrauterine hypoxia or asphyxia. His physical and intellectual development since childhood paralleled that of his peers. Three months ago, the patient experienced unexplained onset of weakness in the right lower limb. This weakness progressively involved the left lower limb and both upper limbs, leading to difficulties in lifting heavy objects, climbing stairs, and rising from a squatting position. Symptoms intensified following physical activity but improved with rest. Throughout the disease course, he did not exhibit ptosis, binocular vision issues, chewing or swallowing difficulties, dyspnea, muscle pain, fasciculations, atrophy, or limb paresthesia. The proband has one younger brother, who has not exhibited any similar symptoms. His father and paternal aunt, who exhibited symptoms from a young age, displayed fluctuating general weakness and recurrent hyperemesis similar to the proband. Due to their deaths in middle age from complications such as severe malnutrition and aspiration pneumonia, no genetic testing could be performed on these paternal relatives.

Cardiopulmonary and abdominal examinations revealed no significant abnormalities. Neurological examination revealed normal high-level cortical functions, no craniopathies, and normal muscle tone in the limbs. Importantly, the patient exhibited none of the additional common signs of Myasthenia Gravis such as ptosis, diplopia, Cogan’s sign, or lid lag. Head-up muscle strength was graded at 5. Strength of the proximal muscles in the upper and lower extremities was rated at 4+, while distal muscle strength was graded at 5. Normoreflexic extremities without extensor toe signs. No cerebellar signs were noted and the sensation to pin prick and joint position sense was intact. The fatigue test was positive for both upper and lower extremities, and the Neostigmine test yielded positive results.

Pertinent diagnostics revealed elevated serum levels: lactate dehydrogenase at 273 U/L (normal range: 120–250 U/L), creatine kinase at 889 U/L (normal range: 50–310 U/L), and creatine kinase isoenzyme at 26.8 U/L (normal: <4.87 ng/ml). All immune, oncological, and metabolic indices fell within normal ranges. The immune evaluation comprised a complete blood count with differential, levels of immunoglobulins, and inflammatory markers including C-reactive protein and erythrocyte sedimentation rate. The oncological evaluation monitored tumor markers pertinent to the clinical scenario. Metabolic assessments were conducted through fasting glucose levels, lipid profiles, and tests for liver and kidney function. Myasthenia gravis antibody tests, including anti-AChR, muscle-specific tyrosine kinase, serum connexin, ryanodine receptor, and low-density lipoprotein receptor-related protein 4 antibodies, were negative. Chest Computed Tomography Scan (CT), brain Magnetic Resonance Imaging (MRI), and cervical spine MRI revealed no significant abnormalities.

**Fig. 1:**
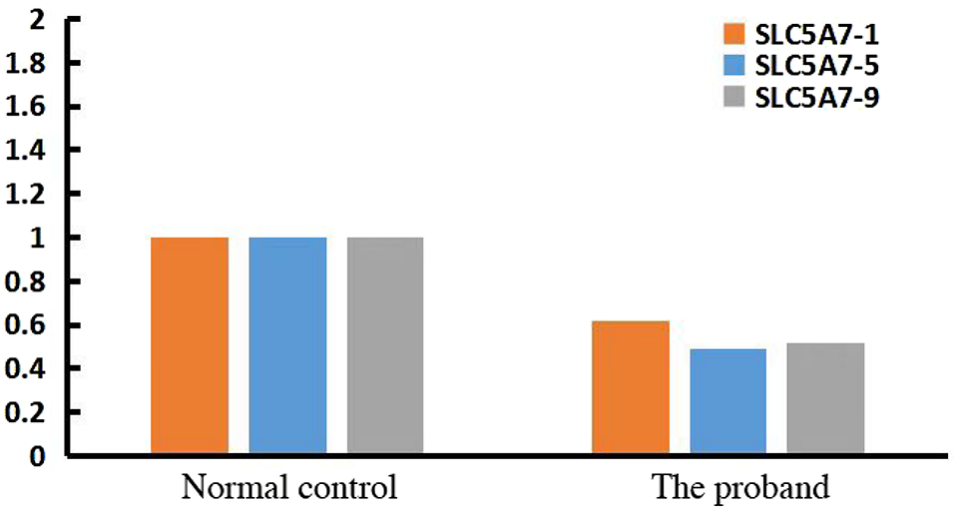
qPCR verification for patient in exon 1, 5, and 9 of the SLC5A7 gene

Nerve electrophysiological examination revealed slowed motor conduction velocities (MCV) in the left median nerve, distal left ulnar nerve, left common peroneal nerve (with prolonged distal latency), and right tibial nerve. Sensory conduction velocity (SCV) tests indicated an absent sensory nerve action potential (SNAP) in the left superficial peroneal nerve and reduced SNAP amplitudes with slowed SCV in the left median, ulnar, and sural nerves. Needle electromyography showed spontaneous potentials in the left gastrocnemius muscle, with no such potentials in other muscles examined. Repetitive nerve stimulation (RNS) tests were positive in the left common peroneal nerve, right tibial nerve, and left axillary nerve (deltoid muscle). (Figure 1). The nasalis nerve was not tested.

**Fig. 2:**
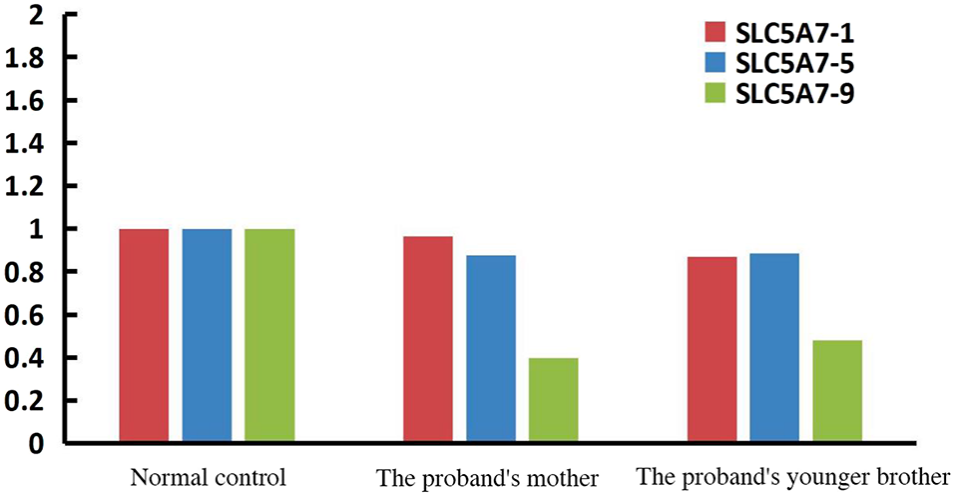
qPCR verification for the patient’s mother and brother in exon 1, 5, and 9 of the SLC5A7 gene

**Fig. 3:**
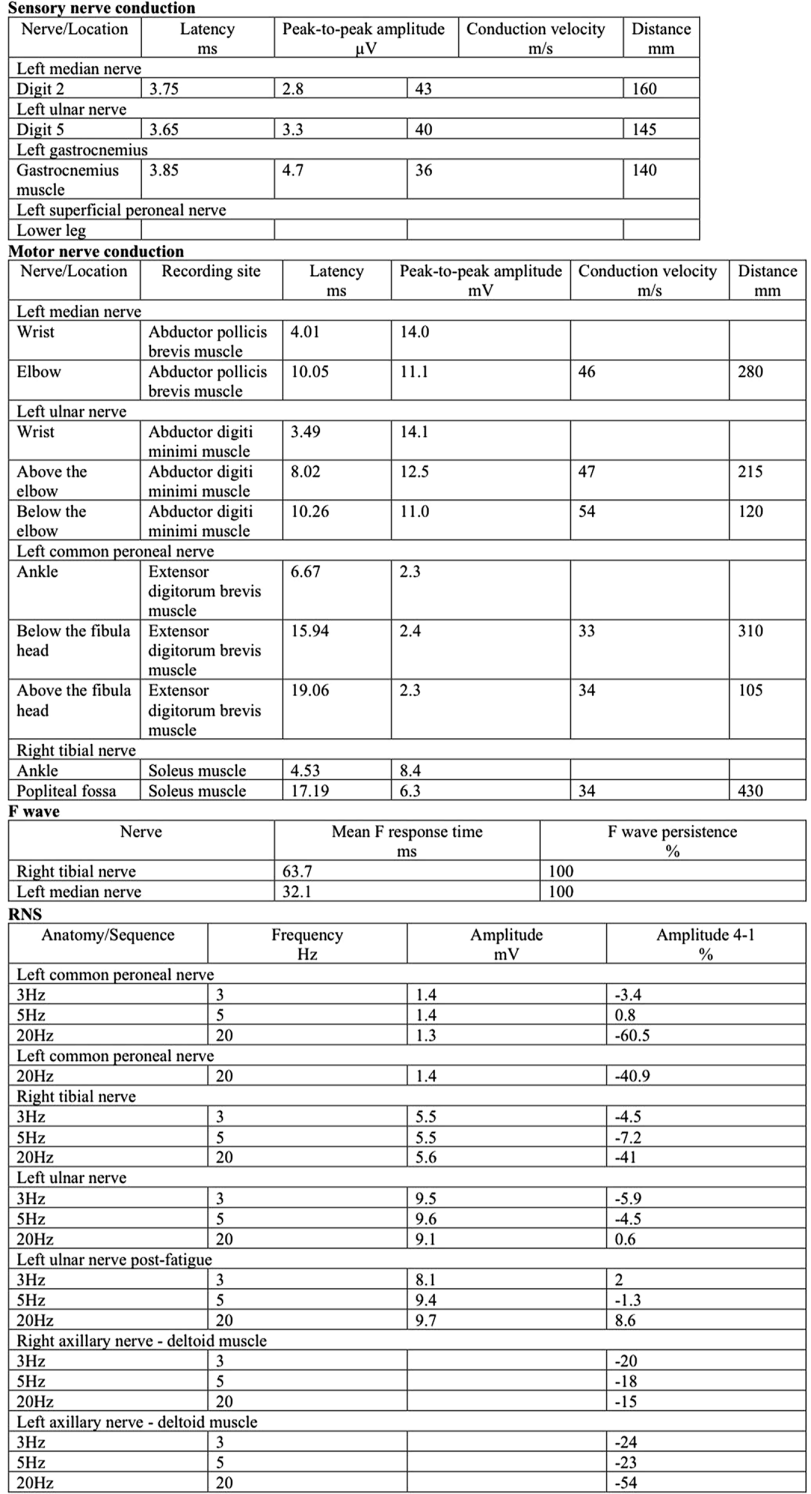
Electromyography report for the patient

Given the patient’s father and aunt had recurrent episodes of similar symptoms prior to their deaths, a genetic basis was considered. Consequently, enhanced second-generation sequencing of the patient’s whole exome was conducted. DNA samples extracted from the patient’s peripheral blood cells underwent Whole Exome Sequencing (WES) at Jiyuan Medical Laboratory. Deletion fragments were then verified using qPCR (Fig. 2). The analysis revealed a heterozygous deletion in exons 1–9 of the SLC5A7 gene, a finding not recorded in the ClinVar database and absent in current literature. As per ACMG standards and guidelines, these variants are classified as “likely pathogenic” (PVS1 + PM2_supporting). Notably, the patient’s father and aunt are deceased, and his mother and younger brother exhibit no phenotypic abnormalities. Consequently, qPCR validation for the patient’s mother and younger brother was also conducted (Fig. 3), indicating a heterozygous deletion of exon 9 in the SLC5A7 gene for both.

Following treatment with pyridostigmine, the patient was discharged with improved proximal muscle strength in the limbs. One month after discharge, a follow-up showed enhanced ability in climbing stairs and rising from a squat compared to prior assessments.

## Discussion

CMS is a rare genetic disorder marked by disrupted neuromuscular transmission. CMS primarily affects neonates and infants, with a minority of cases emerging during adolescence or adulthood. British data indicates a prevalence rate of approximately 9.2 per 1 million children under 18 [4], while adult prevalence rates are not documented. CMS can be classified based on the location of the protein encoded by the mutant gene in the NMJ into presynaptic, synaptic, postsynaptic, and glycosylation defect categories. Mutations in the SLC5A7 gene, which encodes the CHT, result in presynaptic CMS. This condition impairs choline uptake in the presynaptic membrane, affecting the biosynthesis of acetylcholine.

The SLC5A7 gene resides in the 2q12.3 chromosomal region. The CHT, encoded by this gene, comprises 580 amino acids and forms 13 transmembrane domains, including two conserved domains typical of the sugar transport protein family. CHT is transported to the NMJ via axonal transport [5], where it reuptakes choline from the synaptic space into presynaptic nerve terminals, facilitating the synthesis and release of acetylcholine. Genetic testing revealed a heterozygous deletion in exons 1–9 of the SLC5A7 gene (CN = 1) in this patient, affecting the transmembrane region of CHT. This disruption hindered its cell surface localization, causing protein instability and reducing CHT levels on the plasma membrane [6]. The terminal exon of SLC5A7 contains a dileucine endocytic sequence and a secondary sequence, which are crucial for vesicular trafficking and enhancing CHT expression on the membrane surface, respectively [7]. WES identified a large deletion in one chromosome of the patient’s SLC5A7 gene, while the other chromosome was normal. qPCR results indicated that the copy number of the target gene’s exons was approximately half that of a normal individual. Consequently, synthesized CHT could still be transported to the plasma membrane via axons, resulting in a milder clinical phenotype. However, the specific functions of the proteins encoded by different exons of the SLC5A7 gene remain unclear and warrant further investigation.

The proband in this study exhibited symptoms beginning in middle age, characterized by progressively worsening fluctuating weakness and fatigue intolerance. Physical examination showed slightly reduced proximal limb muscle strength, and the fatigue test was positive. Electrophysiological examination plays a crucial role in diagnosis. EMG indicated chronic neurogenic damage with asymmetric involvement, predominantly affecting the left limb. RNS tests were positive in the left common peroneal nerve, right tibial nerve, and left axillary nerve (deltoid muscle). Prior studies on six patients with SLC5A7 gene mutations [8–10] revealed significant reductions in low-frequency RNS in five cases. Only one case demonstrated a decrease in compound muscle action potential (CMAP) following 10 s of high-frequency RNS stimulation. This patient’s RNS demonstrated decreases in both low and high frequencies. Given the SLC5A7 gene variation reduces CHT activity or quantity and choline recovery is a limiting factor in acetylcholine synthesis [11], pathological CHT leads to impaired choline reuptake. This reduces acetylcholine release, lowers muscle fiber endplate potentials below threshold, and diminishes the number of excited muscle fibers, resulting in decreased CMAP amplitude. Additionally, the proband exhibited moderate elevations of creatine kinase in the peripheral blood on several occasions, indicating muscle involvement possibly linked to long-term denervation of muscle fibers.

Notably, besides fluctuating weakness, a significant symptom in the proband’s father and aunt was recurrent severe vomiting, worsening post-infection and fever. This was particularly evident in the aunt, whose symptoms abruptly intensified during pregnancy. CHT is located in the presynaptic membrane, and its function is closely associated with sodium (Na^+^). Sodium ions (Na^+^) facilitate choline uptake and acetylcholine synthesis. An increased influx of Na^+^ or a higher extracellular potassium (K^+^) concentration can amplify choline uptake by CHT. Additionally, choline uptake and acetylcholine release are calcium (Ca^2+^)-dependent processes, but can be inhibited by magnesium (Mg^2+^) [11], Consequently, the efficiency of choline uptake and acetylcholine release is intricately linked to peripheral sodium (Na^+^), potassium (K^+^), and other cation concentrations. In the proband’s family, disease exacerbation was thought to be associated with the body’s stress state. This stress state disrupted electrolyte balance, impairing choline uptake and acetylcholine release, which decreased vagus nerve excitability and led to low-potassium paralytic intestinal obstruction. Recurrent intestinal perforation has been documented in related literature [10]. Thus, it is hypothesized that cholinergic neurotransmission defects may significantly contribute to gastrointestinal motility disorders and gastric perforation in patients.

Given that acetylcholine signaling is prevalent in both peripheral and central cholinergic synapses, mutations in this gene can result in mental illness and cognitive impairment, along with impaired motor function [12].While patients in this study displayed no relevant symptoms, long-term follow-up observation may be necessary.

Mutations in the SLC5A7 gene have been linked to only two clinical phenotypes: VIIa, an autosomal dominant distal hereditary motor neuropathy, and CMS20, an autosomal recessive disorder. The VIIa phenotype predominantly affects motor neurons, manifesting as progressive muscle weakness and atrophy in distal extremities, often with vocal cord paralysis due to vagus nerve involvement [9]. CMS20, on the other hand, may present with infantile onset apnea, cardiac arrest, muscle weakness, intermittent dysphagia, and neurodevelopmental disorders [10]. Distal hereditary motor neuropathy VIIa is caused by a frameshift mutation in the last exon of the SLC5A7 gene, leading to C-terminal truncation of CHT. Consequently, the deletion of exon 9 in this patient’s SLC5A7 gene is predicted to cause CHT protein truncation, potentially resulting in a phenotype akin to distal hereditary motor neuropathy VIIa. While C-terminal truncation mutations significantly reduce CHT activity, patients with distal hereditary motor neuropathy VIIa exhibit a later onset age, milder phenotype, and slower disease progression. In contrast, patients with the autosomal recessive CMS20 typically have an earlier onset age and more severe symptoms, indicating that the impact of truncation mutations on CHT activity is less deleterious than that of autosomal recessive missense mutations. This patient displayed an adult-onset autosomal dominant CMS, a phenotype not previously reported in literature, meriting heightened clinical attention. Importantly, differential diagnosis should consider immune and metabolic diseases, mitochondrial disorders, and others.

The disease phenotype resulting from SLC5A7 gene mutations is notably complex. Currently, the limited number of cases restricts our understanding, but this will expand as more cases are identified. The correlation between genotype and phenotype will become clearer over time, potentially leading to greater recognition of SLC5A7 gene-related spectrum diseases. A comprehensive exploration of the genotype-phenotype correlation is crucial for the accurate treatment of genetic diseases. This paper is the first to report that a heterozygous deletion of exons 1–9 in the SLC5A7 gene causes CMS, thereby enriching our understanding of the gene’s genetic and phenotypic spectrum. Additionally, this is the first documented case of CMS in China caused by SLC5A7 gene variation, broadening the geographical distribution of mutations in this gene.

## Data Availability

The datasets generated and/or analyzed during the current study are available in the NCBI repository, reference number [BioProject: PRJNA1086838 BioSample: SAMN40413909 SRA: SRR28300810].

## Abbreviations

ACMG: American college of medical genetics and genomics
CHT: Choline transporter
RNS: Repetitive nerve stimulation
NMJ: Neuromuscular junction
MCV: Motor conduction velocity
SCV: Sensory conduction velocity
SNAP: Sensory nerve action potential
CMAP: Compound muscle action potential
CT: Computed tomography scan
MRI: Magnetic resonance imaging
